# Non-Thermal Plasma-Driven Degradation of Organic Dyes Using CeO_2_ Prepared by Supercritical Antisolvent Precipitation

**DOI:** 10.3390/nano15231831

**Published:** 2025-12-04

**Authors:** Qayam Ud Din, Maria Chiara Iannaco, Iolanda De Marco, Vincenzo Vaiano, Giuseppina Iervolino

**Affiliations:** Department of Industrial Engineering, University of Salerno, Via Giovanni Paolo II, 132, 84084 Fisciano, SA, Italy; qdin@unisa.it (Q.U.D.); idemarco@unisa.it (I.D.M.); vvaiano@unisa.it (V.V.)

**Keywords:** non-thermal plasma, CeO_2_ nanocatalyst, supercritical antisolvent synthesis, plasma–catalyst synergy, advanced oxidation processes, wastewater treatment

## Abstract

Non-thermal plasma (NTP) is a fast, reagent-free technology for dye removal, yet its performance is highly dependent on the operating conditions and on plasma–catalyst interactions. In this work, a coaxial falling-film dielectric barrier discharge (DBD) reactor was optimized for the degradation and decolorization of organic dyes, with ceria (CeO_2_) employed as a catalyst. For the first time, CeO_2_ prepared via a supercritical antisolvent (SAS) micronization route was tested in plasma-assisted dye decolorization and directly compared with its non-micronized counterpart. Optimization of plasma parameters revealed that oxygen feeding, an input voltage of 12 kV, a gas flow of 0.2 NL·min^−1^, and an initial dye concentration of 20 mg·L^−1^ resulted in the fastest decolorization kinetics. While the anionic dye Acid Yellow 36 exhibited electrostatic repulsion and negligible plasma–ceria synergy, the cationic dyes Crystal Violet and Methylene Blue showed strong adsorption on the negatively charged CeO_2_ surface and pronounced plasma–catalyst synergy, with SAS-derived CeO_2_ consistently outperforming the non-micronized powder. The SAS catalyst, characterized by a narrow particle size distribution (DLS) and spherical morphology (SEM), ensured improved dispersion and interaction with plasma-generated species, leading to significantly shorter decolorization radiation times compared to the literature benchmarks. Importantly, this enhancement translated into higher energy efficiency, with complete dye removal achieved at a lower specific energy input than both plasma-only operation and non-micronized CeO_2_. Scavenger tests confirmed •OH radicals as the dominant oxidants, while O_3_, O_2_•^−^, and e^−^_a_ played secondary roles. Tests on binary dye mixtures (CV + MB) revealed synergistic decolorization under plasma-only conditions, and the CeO_2_-SAS catalyst maintained high overall efficiency despite competitive adsorption effects. These findings demonstrate that SAS micronization of CeO_2_ is an effective material-engineering strategy to unlock plasma–catalyst synergy and achieve rapid, energy-efficient dye abatement for practical wastewater treatment.

## 1. Introduction

Wastewater containing dyes is a serious problem for both the environment and human health. Even at low levels, dyes can block sunlight in rivers and lakes, stopping photosynthesis and harming aquatic life [1]. Some dyes can also break down into harmful substances, like aromatic amines, which are toxic or cancer-causing [2,3]. Many studies have reported that dyes are difficult to remove using natural processes, and they are found in high amounts (often 10–50 mg/L or more) in wastewater from textile industries [4,5].

Many treatment methods are available, like coagulation, adsorption, membrane filtration, biological treatment, electrochemical oxidation, and advanced oxidation processes (AOPs) such as UV/H_2_O_2_ [6,7], but each method has its own drawbacks. Some processes create extra sludge, while others use a lot of energy or chemicals, or do not work well for all types of dyes. Newer reviews suggest that we need fast, reliable methods that work at room temperature, do not need extra chemicals, and can remove even hard-to-degrade dyes [8]. From an energy and cost perspective, UV/H_2_O_2_ and related UV-based AOPs are generally more energy-efficient for low-strength, optically clear effluents, but their performance is strongly affected by solution absorbance and they require continuous chemical dosing and lamp maintenance. Non-thermal plasma, instead, can treat highly coloured wastewater without external oxidants and in compact reactor configurations, albeit often at higher specific energy consumption. In this context, optimizing NTP operation and exploiting plasma–catalyst synergy is essential to approach the energy performance of UV-based processes while retaining the ability to handle strongly absorbing dye effluents [9].

Non-thermal plasma (NTP) is a new and promising solution [10]. It works by creating energetic electrons that form strong oxidants (like •OH, ozone, atomic oxygen, and hydrogen peroxide) directly in the water without adding any chemicals. These oxidants can break down dyes quickly [11]. In DBD (dielectric barrier discharge) reactors, this process can be adjusted by changing the gas used, voltage, frequency, and how the gas and liquid interact [12]. Recent studies have shown that plasma can remove colour fast, and that things like falling-film designs (where water flows in a thin layer) improve the treatment efficiency [13]. In related work, Iervolino et al. (2020) showed that adding H_2_O_2_ to a DBD plasma system helped remove azo dyes more effectively, showing that adjusting the oxidants can make a big difference [14].

Using a catalyst with plasma (called “plasma-catalysis”) can also improve the process. A good catalyst can pull the dye molecules to its surface and help convert slower oxidants (like ozone and H_2_O_2_) into faster ones (like •OH radicals). This improves both the speed and energy efficiency of the process [15]. Among many catalysts, ceria (CeO_2_) stands out because of its unique ability to switch between Ce^3+^ and Ce^4+^ and create oxygen vacancies [16]; this helps with electron transfer and forming reactive radicals. New studies show that modified ceria (with more surface defects) can degrade dyes faster.

However, researchers still have different opinions on how plasma works best. Some say that •OH radicals do most of the work, while others believe ozone is more important, depending on the water’s pH and setup [17]. Also, using a catalyst does not always guarantee better results; if the dye and catalyst have the same charge, they may repel each other, and the dye will not attach to the surface. But if their charges are opposite, the dye sticks well, and the catalyst helps a lot [18]. In real wastewater, things are even more complex. Sometimes two dyes can compete with each other for the same active sites, slowing things down [9]. But in some cases, they can also help each other degrade faster, which has been seen in recent plasma studies using mixed dyes.

However, the overall efficiency of this hybrid system largely depends on the properties of the photocatalyst. To maximize its performance, the photocatalyst requires a high surface area, controlled particle size, and optimized morphology, which promote charge separation and reduce recombination [19]. The Supercritical Antisolvent (SAS) process offers a sustainable and versatile approach to producing nanostructured photocatalysts with tailored characteristics that are hard to achieve with traditional methods [20].

Recent studies on CeO_2_-based catalysts for dye decolorization have shown that their performance can be significantly improved by carefully controlling particle size and morphology, by creating oxygen vacancies and other surface defects that facilitate Ce^3+^/Ce^4+^ redox cycling, by introducing suitable dopants, and by dispersing CeO_2_ on high-surface-area supports to enhance the accessibility of active sites. Various wet-chemical routes such as co-precipitation, sol-gel, and hydrothermal synthesis can effectively regulate particle size, dispersion, and surface charge. The supercritical antisolvent (SAS) route adopted here provides a complementary, sustainable approach that combines narrow size distribution, high dispersion, and tunable surface properties in a single step, which is particularly attractive for maximizing contact between plasma-generated reactive species and the catalyst surface [21].

By enabling the controlled synthesis of advanced materials, SAS can play a crucial role in unlocking the full potential of plasma-assisted photocatalysis. While ceria has been studied in photocatalysis, no report has yet explored SAS-prepared CeO_2_ in plasma-assisted dye decolorization. This approach allows for tailoring particle size, dispersion, and surface charge to maximize plasma–catalyst synergy [22].

This work aims to benchmark the performance of plasma-only decolorization against plasma-assisted processes in the presence of CeO_2_ catalysts, focusing on the treatment of Acid Yellow, Methylene Blue, and Crystal Violet dyes. The influence of key operational parameters—including gas composition, gas flow rate, applied voltage, and initial dye concentration—is systematically investigated. Particular attention is devoted to comparing non-micronized CeO_2_ with CeO_2_ synthesized via supercritical antisolvent (SAS) precipitation, a material introduced here for the first time in plasma-assisted dye decolorization. The distinct physicochemical features of the SAS-derived catalyst are rationalized through zeta potential measurements and dark adsorption tests. The contribution of reactive species is elucidated via scavenger experiments, and this study is extended to binary dye mixtures to better reproduce realistic wastewater conditions.

The ultimate goal is to identify a practical operating window and to establish a simple yet general mechanistic guideline based on charge matching between the catalyst surface and the dye species in order to unlock and control the plasma–ceria synergy.

## 2. Materials and Methods

### 2.1. Materials

Cerium (III) acetylacetonate hydrate (Ce(acac)_3_, CAS 206996-61-4) was supplied by Thermo Scientific Chemicals (Segrate, Italy). Ethyl alcohol (purity 99.9%) was purchased from Carlo Erba Reagenti (Cornaredo, Italy). CO_2_ at 99% purity was purchased by Morlando Group s.r.l. (Naples, Italy). Acid Yellow 36 (AY36, CAS 587-98-4), Crystal Violet (CV, CAS 548-62-9), and Methylene Blue (MB, CAS 122965-43-9) were supplied by Sigma Aldrich (USA).

### 2.2. SAS Experimental Setup and Test Procedure

**Figure 1:**
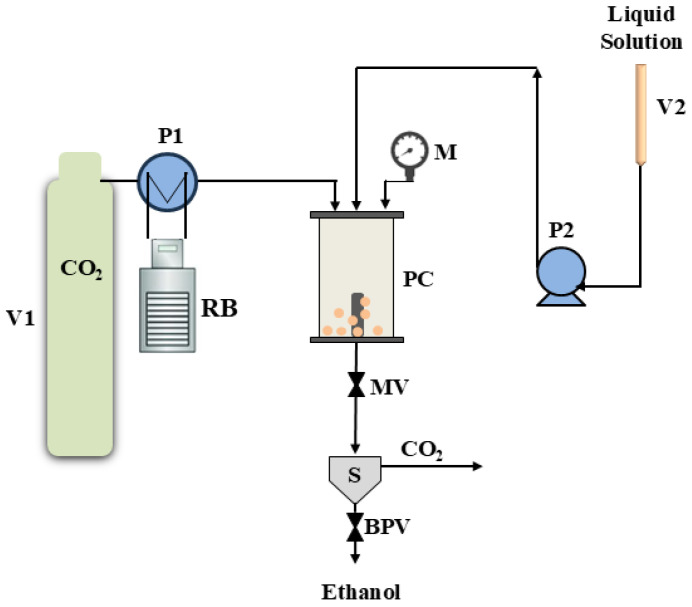
Schematic diagram of the Supercritical Antisolvent (SAS) Set-up: BPV: backpressure valve, M: manometer, MV: micrometric valve, P1: pump of CO_2_, P2: liquid pump, PC: precipitation chamber, RB: refrigerating bath, V1: CO_2_ tank, V2: liquid reservoir.

The Ce(acac)_3_ powder was micronized using the SAS plant represented in Figure 1. A typical SAS precipitation starts by pumping CO_2_ into the precipitator to reach the selected pressure. A specific volume of the liquid solvent is then fed into the chamber to produce the liquid solution’s quasi-steady state condition. A stainless-steel nozzle with a diameter of 100 µm is used to feed the liquid solution after steady-state conditions have been achieved. The liquid phase and CO_2_ are fed by two high-pressure pumps. After the precipitation step, a washing step is necessary to remove any remaining solvent. Then, the precipitated particles are collected using a cylindrical porous filter (pore diameter: 0.1 μm). The conditions optimized in a previous study [23] were applied in this work for the micronization of Ce(acac)_3_, which included a temperature of 40 °C, a pressure of 150 bar, and a total solute concentration of 5 mg/mL. The solvent used was ethanol. The precipitated powder was collected at the end of the experiment, characterized, and used in the photocatalytic tests, after a subsequent calcination step.

### 2.3. Photocatalysts Preparation

The micronized precursor must undergo a subsequent calcination step because the SAS process does not lead to the direct formation of CeO_2_. For both samples (micronized with SAS and not micronized), a slow calcination procedure was used, raising the temperature by 2 °C/min up to 450 °C, and then maintaining it there for two hours [23]. The catalyst produced from the processed precursor (CeO_2_ SAS) and the catalyst produced following the calcination of the non-micronized precursor (CeO_2_ NM) were compared for photocatalytic performance.

### 2.4. Characterization Methods

A field emission scanning electron microscope (FESEM, model LEO 1525, Carl Zeiss SMT AG, Oberkochen, Germany) was used to analyse the samples’ morphology. The powdered materials were dispersed on a carbon tab and coated with a 250 Å gold layer to improve conductivity. The particle size distribution of the suspended photocatalysts was evaluated by performing a DLS analysis using a Zetasizer Nano ZS (Malvern Instruments Ltd., Worcestershire, UK). Solutions in distilled water with a concentration of 1 mg/mL and sonicated for 30 min were used. Using a zeta potential analyser, the zeta potential of photocatalysts was measured. For each measurement, 10 mg of sample powder was dispersed in 10 mL of distilled water by sonication for 30 min. To find the isoelectric point (IEP), the suspension’s pH was adjusted between 5 and 11 by adding small amounts of HCl or NaOH solutions. At each pH, three measurements were recorded, and the average zeta potential was plotted against pH. Infrared spectra were obtained with a Fourier Transform (FT-IR) spectrometer (Alpha model, Bruker Optics, Coventry, UK). For sample preparation, 100 mg of KBr was mixed with 1 mg of the sample to create an infrared-transparent pellet. Measurements covered a wavenumber range of 4000–400 cm^−1^. Ultraviolet-visible diffuse reflectance spectra (UV–Vis DRS) were recorded using a Perkin Elmer Lambda 35 spectrophotometer (Waltham, MA, USA) with an RSA-PE-20 reflectance accessory (Labsphere Inc., North Sutton, NH, USA). Band gap energies were derived from the Kubelka–Munk function (F(R∞)) by plotting [F(R∞) × hν]^0.5^ versus hυ (eV). Raman spectra were collected at room temperature with a Dispersive MicroRaman spectrometer (Renishaw, Wotton-under-Edge, Gloucestershire, UK), using a 514 nm laser over a Raman shift range of 200–1000 cm^−1^. The Brunauer, Emmett, and Teller (BET) specific surface area (SSA) was measured via nitrogen adsorption at −196 °C on a Costech Sorptometer 1042 (Milan, Italy). Before measurement, samples were pretreated at 150 °C for 180 min under helium flow.

### 2.5. Non-Thermal Plasma Experimental Setup

**Figure 2:**
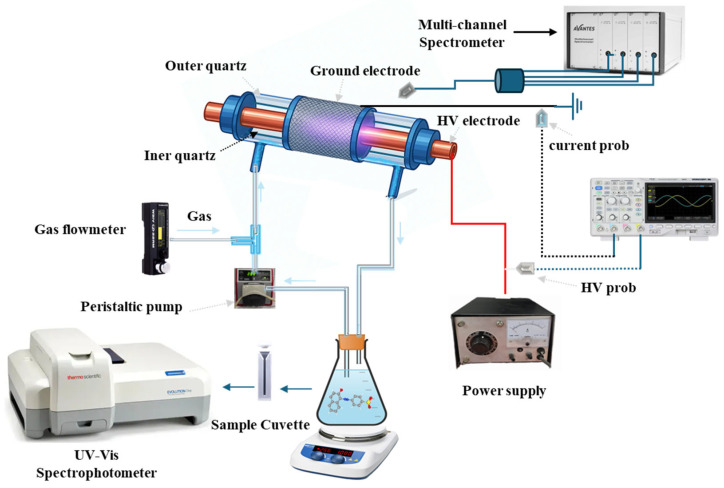
Experimental setup for non-thermal plasma DBD reactor.

A cylindrical dielectric barrier discharge (DBD) reactor was used for the experimental tests, as shown in Figure 2. The reactor was inclined at an angle of 30° to maintain a uniform flow of wastewater through the quartz tube containing the electrodes. The system consisted of two coaxial quartz tubes. The inner quartz tube had an inner diameter of 9 mm with a wall thickness of 1.5 mm. Inside this tube, a copper high-voltage electrode was placed, designed as a small cylindrical tube with a diameter of 8 mm and a length of 200 mm. The outer quartz tube was 200 mm in length, with an inner diameter of 24 mm and a wall thickness of 1.5 mm. A stainless-steel mesh, serving as the grounded electrode, was wrapped around the outer quartz tube, covering a length of 90 mm. Plasma was generated in the discharge region between the two electrodes, separated by the quartz dielectric, thereby establishing the DBD configuration. Figure S1 shows the real time picture of plasma reactor used in this study.

The dye-containing solution was stored in a Pyrex tank, and a peristaltic pump was used to uniformly circulate the solution between the DBD reactor and the external tank. The liquid flow rate was maintained at 80 mL/min throughout the experiments. During experiments, the contaminated water continuously flowed between the reactor and the tank, forming a thin film along the inner surface of the outer quartz tube. In this configuration, the plasma generated between the electrodes directly interacted with the thin water film, producing reactive plasma species responsible for the decolorization of the contaminants. For plasma generation, a feeding gas was required. Oxygen and air were used as the feed gases, with flow rates ranging from 0.1 to 0.3 NL/min. During the experiments in presence of catalyst, a specific amount of CeO_2_ (non-micronized (NM) or synthesized via SAS procedure) equal to 0.05 g was used.

The electrical characteristics of the DBD reactor were measured using a Tektronix P6015A (Italy) high-voltage probe and a Tektronix P6021A (Italy) current probe, connected to a Yokogawa DLM3022 (Italy) oscilloscope operating in DC coupling. A 0.47 µF reference capacitor was inserted in series with the reactor, and its voltage was monitored using a Tektronix P6139A (Italy) probe to evaluate charge transfer. Under these conditions, the loop area was 1.15 × 10^−3^ J per cycle, corresponding to an average discharge power of 23 W.

### 2.6. Analytical Measurements

To evaluate the decolorization of Acid Yellow 36 and Crystal Violet with and without the ceria catalyst under non-thermal plasma treatment, the analysis of the liquid phase was performed. UV–visible absorption spectra of the liquid samples were recorded at different time intervals using a Thermo-Fisher Evolution 201 (Italy) spectrophotometer, in the wavelength range of 200–800 nm.

Optical emission spectroscopy (OES) was used as a non-intrusive diagnostic to characterize the discharge. Plasma emission spectra were collected through the quartz tube, with the Fiber-optic probe positioned approximately 3 mm from the outer surface in the active plasma region and analysed using a multichannel high-resolution spectrometer (AVASPEC-4DT, Avantes (The Netherlands)) covering 200–900 nm. The system comprises four AVASPEC-ULS4096CL-EVO channels equipped with 1800/0.5 gratings, providing spectral ranges of 200–367 nm, 360–506 nm, 500–624 nm, and 620–900 nm. Spectra were recorded at the optimized operating conditions for both water only and Oxygen with water. The resulting emission spectra are reported in Figure S7.

The plasma was driven by an AC power supply PVM500/DIDRIVE10 (UNLIMITED) (USA) delivering a sinusoidal waveform at 20 kHz with a peak voltage of 12 kV Figure S2a. The average discharge of power was obtained over one period *T* was calculated as follows:

$$P=\;\frac{1}{T}\;\int_{0}^{T}v(t)i(t)dt$$

As shown in Figure S2b the energy per cycle was also determined from the Lissajous (Q–V) figure using:

$$Q=C_{ref}*V_{cap}$$

According to Beer–Lambert’s law, the absorbance is directly proportional to the concentration of the dye in the solution. Based on this principle, the maximum absorbance peaks were monitored at 436 nm for Acid Yellow 36 and 590 nm for Crystal Violet and 663 nm for Methylene Blue.

The decolorization efficiency was calculated as follows:

$$Degradation\;efficiency=(1-\frac{C}{C_{o}})\times 100$$

where:

C = dye concentration after the treatment;

C_o_ = initial dye concentration when time is zero.

In addition, the efficiency of the DBD plasma reactor was expressed in terms of energy yield, which is defined as the amount of dye decomposed per unit of energy consumed during the process. This parameter provides a direct measure of the reactor’s energy efficiency in pollutant decolorization which is expressed in g/kWh.

$$Y=\frac{C_{o}\cdot V\cdot conv(\%)}{P\cdot t}$$

Co is the initial concentration of dye (g/L). V is the volume of solution (L). P is the input power (kW). Conv (%) is the dye decolorization in % and t (h) the time required for the decolorization of dyes.

Following equation is used to calculate the first order kinetic constant and half-life for the decolorization of the CV and AY36 dyes.

$$-\ln\left(\frac{C}{C_{o}}\right)=K_{d}\cdot t$$

t12=ln2kd

where $K_{d}$ is the decolorization apparent kinetic constant, t12 half-life and t is the treatment time.

All key experiments were carried out three times independently (n = 3). The reported values correspond to the arithmetic mean, and the associated error bars represent the standard error of the mean. Data handling and plotting were performed using Microsoft Excel (Microsoft 365) and Origin-Pro (9.0). Statistical differences between selected data sets were assessed by Student’s *t*-test, adopting a significance level of *p* < 0.05. Statistically significant differences are marked in the figures as * (*p* < 0.05), ** (*p* < 0.01), and *** (*p* < 0.001).

## 3. Results and Discussion

### 3.1. Catalyst Characterization Results

#### 3.1.1. Physicochemical Characterizations

**Figure 3:**
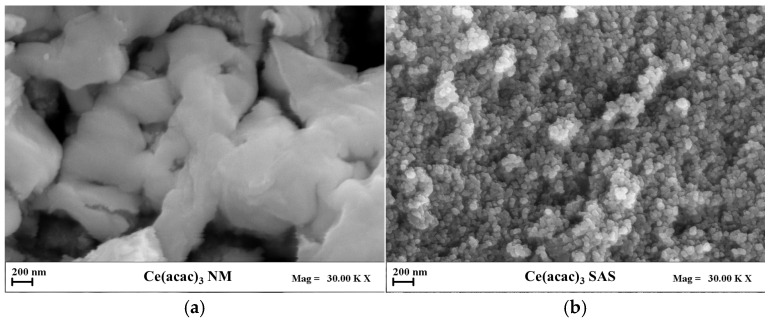

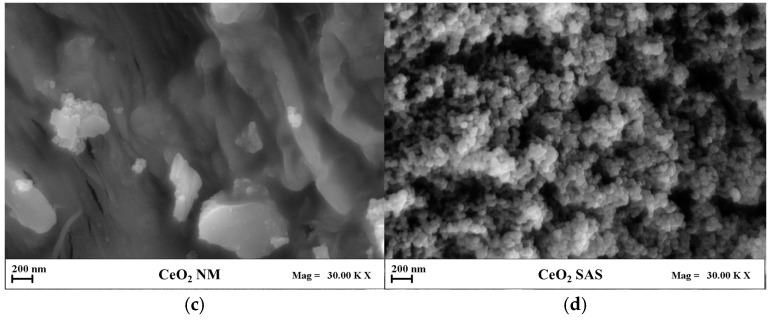
FESEM images of precursors (**a**) non-micronized Ce(acac)_3_, (**b**) SAS-micronized Ce(acac)_3_, (**c**) non-micronized CeO_2_, (**d**) CeO_2_ obtained from the precursor micronized by the SAS process.

The FESEM images of the unprocessed and SAS-micronized precursors are shown in Figure 3a,b, respectively. A marked morphological difference can be observed: while the unprocessed Ce(acac)_3_ precursor (Figure 3a) consists of large, compact aggregates, the SAS-treated sample (Figure 3b) exhibits nanometric, well-dispersed spherical particles, confirming the effectiveness of the supercritical antisolvent process in reducing particle size. After calcination, both materials retained their original morphology (Figure 3c,d). The CeO_2_ NM sample (Figure 3c) preserved its aggregated structure, whereas CeO_2_ SAS (Figure 3d) maintained the fine, uniform texture derived from SAS precipitation, suggesting that the thermal treatment did not alter the nanoscale organization of the SAS-derived particles.

**Figure 4:**
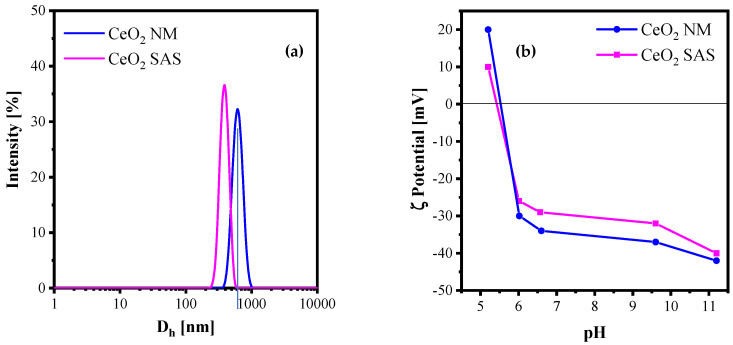
(**a**) Hydrodynamic diameter (D_h_) of CeO_2_ NM and CeO_2_ SAS; (**b**) Isoelectric point (IEP) of CeO_2_ NM and CeO_2_ SAS.

Since the catalytic performance depends not only on the specific surface but also on its behavior in the aqueous reaction medium, both BET surface area measurements and dynamic light scattering (DLS) analyses were performed, with the results summarized in Table 1. The data indicate that the SAS process yields a catalyst with a specific surface area comparable to that of the non-micronized material. However, BET analysis reflects the total accessible surface area under dry conditions, whereas DLS provides insight into particle dispersion, aggregation state, and hydrodynamic diameter in suspension—parameters that are critical for catalytic efficiency in liquid-phase processes. Comparison between the SAS-derived CeO_2_ (composed of nanosized primary particles) and the non-micronized CeO_2_ (formed by larger, irregular fragments) reveals a significant difference in dispersibility after sonication (Figure 4a). Specifically, CeO_2_ SAS exhibited a hydrodynamic diameter of 387 nm, whereas CeO_2_ NM displayed a value of 618 nm under identical dispersion conditions. These differences originate from distinct interparticle cohesion mechanisms: nanoparticle agglomerates are held together mainly by weak van der Waals forces over small contact areas, making them mechanically fragile and easily disrupted by cavitation during ultrasonication. In contrast, the larger, irregular fragments of CeO_2_ NM establish stronger and more extensive interparticle contacts reinforced by mechanical interlocking, which renders them less susceptible to sonication-induced fragmentation.

The isoelectric point (IEP) of the two photocatalysts was determined by measuring their zeta potential over a range of pH values (Figure 4b). This analysis provides valuable information on the surface charge behavior of the particles under the experimental conditions, which strongly influences dye adsorption on the catalyst surface. Knowledge of the IEP enables prediction of whether the catalyst surface is positively or negatively charged at a given pH, thereby controlling the electrostatic interactions with dye molecules and, consequently, the overall catalytic removal efficiency [24]. For both catalysts, the isoelectric point was in the range 5.4–5.6.

#### 3.1.2. Spectroscopic Characterizations

**Figure 5:**
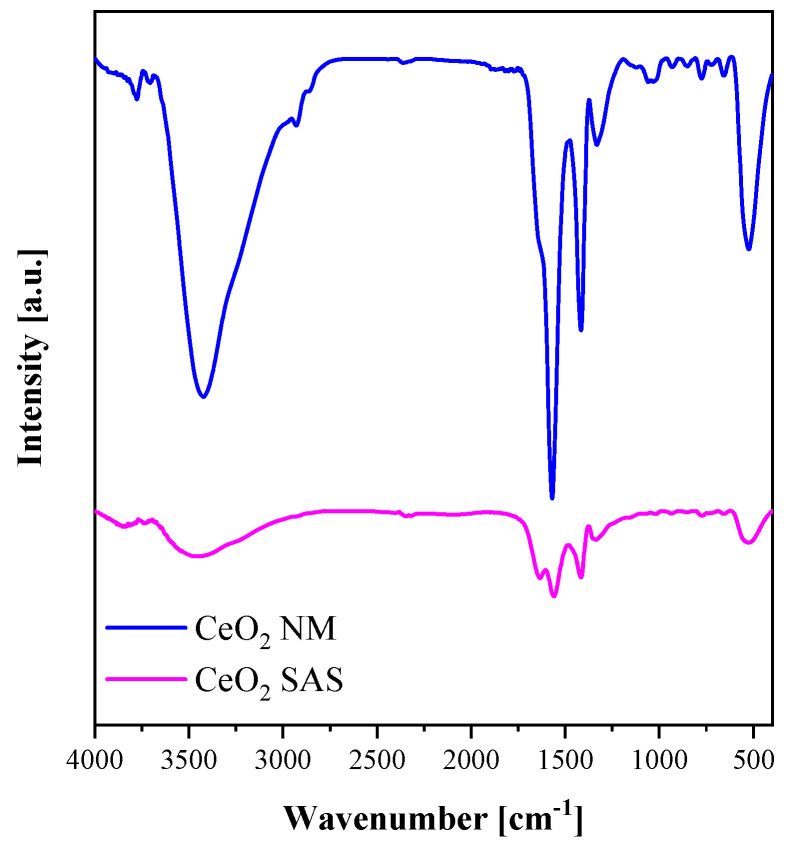
FT-IR spectra of CeO_2_ SAS and CeO_2_ NM catalysts.

Several techniques, including FT-IR spectroscopy, Raman spectroscopy, and UV–Vis diffuse reflectance spectroscopy, were employed to characterize the catalysts obtained after calcination of both the non-micronized and SAS-processed precursors. Figure 5 presents the FT-IR spectra of all CeO_2_ samples. In all cases, a broad absorption band cantered around 3430 cm^−1^ is attributed to the O–H stretching vibrations of physiosorbed water molecules on the CeO_2_ surface [25]. The bands observed at 1410 cm^−1^ and 1560 cm^−1^ are associated with carbonate-like species, corresponding to coordinated CO_2_ and physically adsorbed CO_2_, respectively [26]. The signal at approximately 1620 cm^−1^ arises from the bending vibration of surface hydroxyl groups [27], whereas the band near 510 cm^−1^ is characteristic of Ce–O stretching modes, related to the vibrations of oxygen atoms bound to cerium within the crystal lattice [28]. By comparing the intensity of the OH stretching vibration (~3430 cm^−1^) with that of the Ce-O lattice vibration band (~510 cm^−1^), the ratio results slightly higher for the SAS sample, suggesting a higher amount of surface hydroxyl groups [29] (Table 2).

**Figure 6:**
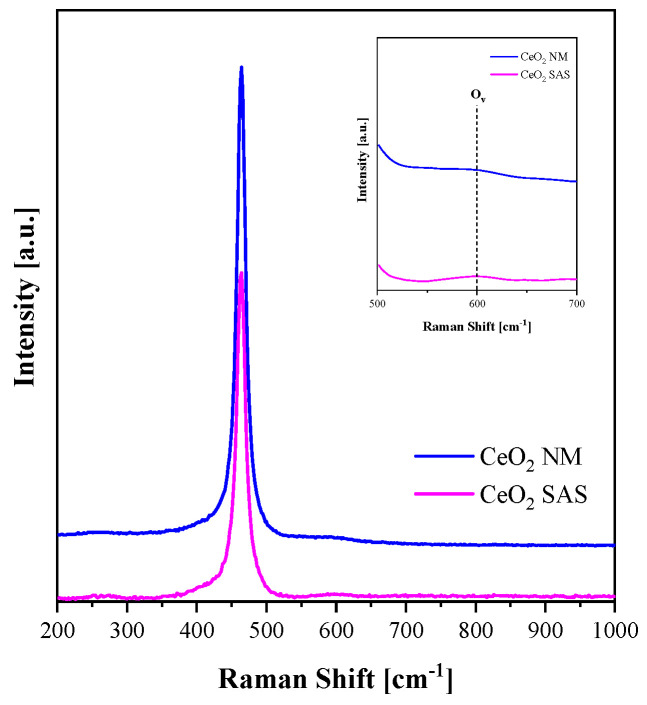
Raman spectra of CeO_2_ SAS and CeO_2_ NM catalysts.

Figure 6 displays the Raman spectra of the CeO_2_ catalysts in the 200–1000 cm^−1^ range. The prominent peak at 462 cm^−1^ corresponds to the F_2_g symmetric vibrational mode of oxygen atoms surrounding Ce^4+^ ions in the fluorite lattice of CeO_2_, characteristic of its long-range crystalline order [30]. A weaker band observed at approximately 607 cm^−1^ is typically associated with oxygen vacancies (O_V_) within the ceria lattice. The intensity ratio between the O_V_ and F_2_g peaks is commonly used to estimate the relative concentration of oxygen vacancies, with higher ratios indicating greater defect densities [31]. Such Ov can act as active sites for the adsorption of oxygen and water molecules, thereby promoting the formation of reactive oxygen species that enhance catalytic activity [32]. In the present study, the SAS-derived CeO_2_ exhibited a slightly higher O_V_/F_2g_ ratio compared to the non-micronized sample (Table 2), suggesting that the SAS process may favor the generation of structural defects beneficial for catalytic performance.

**Figure 7:**
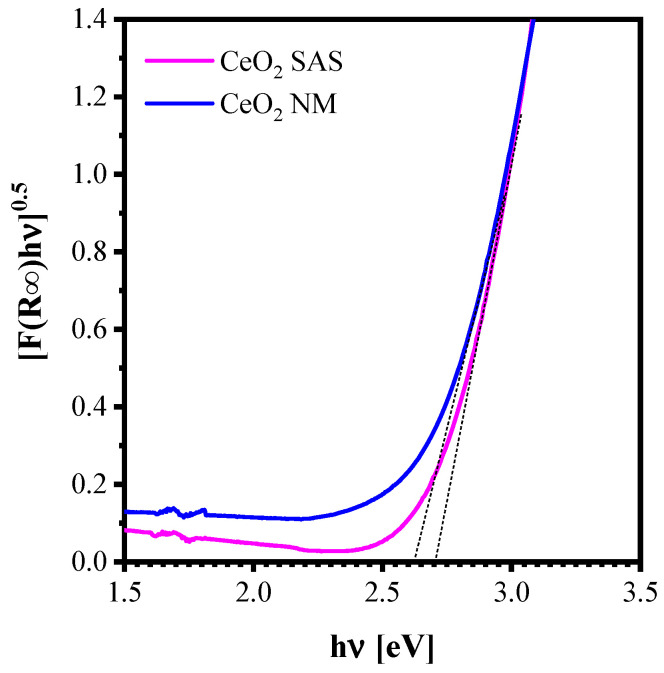
Tauc plot of CeO_2_ SAS and CeO_2_ NM catalysts.

The optical band gap energy of the catalysts was estimated from the UV–Vis diffuse reflectance spectra by applying the Kubelka–Munk function, plotting [F(R∞) × hv]^0.5^ vs. hν (Figure 7). The calculated band gap values are reported in Table 2. Both CeO_2_ catalysts exhibited similar band gap energies, indicating that the SAS micronization process did not significantly alter the optical properties of the material.

### 3.2. Optimization of Only-Plasma Operating Parameters for Acid Yellow 36 Decolorization

#### 3.2.1. Effect of Feeding Gas

**Figure 8:**
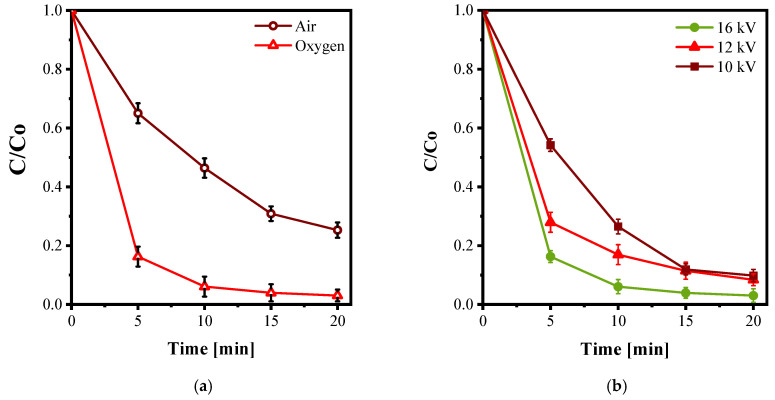
Effect of different feeding gasses (**a**) and effect of different input voltages (**b**), at 20 kHz, Q_gas_: 0.2 NL m^−1^, AY36 initial concentration: 20 mg/L.

The choice of feeding gas strongly influences AY36 decolorization, as it determines both the type and the concentration of reactive species generated by the plasma. As shown in Figure 8a, oxygen markedly outperformed air: after only 5 min of treatment, more than 80% decolorization was achieved with O_2_, compared to approximately 37% with air. This result aligns with the established understanding that O_2_-fed DBD systems produce a richer flux of oxidative species such as atomic oxygen (O), hydroxyl radicals (•OH), ozone (O_3_), and hydrogen peroxide (H_2_O_2_). In contrast, when air is used as the feed gas, the presence of N_2_ promotes the formation of reactive nitrogen species (RNS), which can compete with or quench reactive oxygen species (ROS), thus reducing the overall oxidation strength. The progressive decolorization of AY36 can also be observed in the absorbance spectra reported in Figure S3b. These findings are consistent with modeling and diagnostic studies that have documented analogous shifts in ROS/RNS distributions with gas composition in DBD water reactors [33].

The decolorization kinetics of AY36 followed a pseudo-first-order model (Table 3). The O_2_ plasma exhibited a significantly higher apparent rate constant (k_d_) and shorter half-life (t_1/2_) than the air plasma, confirming its superior oxidative efficiency. Similar trends have been reported by Huang et al. (2024), who compared O_2_-DBD and air-DBD configurations in a bubbling-array reactor for various dyes (sunset yellow, methyl orange, and methyl violet), observing faster kinetics and higher removal efficiencies (~99–100% in 20 min under O_2_), with •OH and •O_2_^−^ identified as the main oxidants [34]. Likewise, Attri et al. (2018) investigated the effect of gas composition in plasma-assisted dye decolorization and reported enhanced decolorization when oxygen was present, due to a favorable ROS/RNS balance [35]. These results collectively confirm that the use of oxygen as the feeding gas provides a more oxidative plasma environment, enabling faster and more efficient AY36 decolorization.

More broadly, recent reviews on non-thermal plasma applications in water treatment have emphasized that the gas composition is a key operational parameter, as it governs the ROS/RNS balance and consequently the decolorization pathways and reaction rates. While air-fed DBD systems can still achieve effective pollutant removal, oxygen or oxygen-enriched feeds typically enhance ROS generation—particularly ozone (O_3_) and hydroxyl radicals (•OH)—and thereby accelerate decolorization [10], in full agreement with the behavior observed in this study.

#### 3.2.2. Effect of Input Voltage

The input voltage determines the electric field strength within the DBD gap and thus controls the generation of energetic electrons, their collision frequency with gas molecules, and the subsequent formation of reactive species such as O, •OH, O_3_, and H_2_O_2_. As shown in Figure 8b, higher applied voltages resulted in faster AY36 decolorization after 5 min: 83% at 16 kV, 71% at 12 kV, and 53% at 10 kV. However, at 16 kV, the discharge became unstable and filamentary, with visible hot spots on the quartz surface, whereas at 10 kV the plasma was weak and decolorization slower. The experimental data (Table 4) followed pseudo-first-order kinetics at all voltages, with the apparent rate constant and the corresponding t_1/2_ decreasing as the voltage was raised. The best compromise between plasma stability and decolorization efficiency was observed at 12 kV, which was therefore selected for subsequent experiments.

This trend is consistent with previous reports. Wu et al. (2019) studied methylene blue decolorization in a DBD reactor and observed that higher voltages enhance decolorization rates, as stronger electric fields generate larger amounts of active species at the gas–liquid interface [36]. Similarly, Guo et al. (2022) reported a substantial improvement in tetracycline removal efficiency—from 26% at 5 kV to 83–85% at 7–9 kV—directly confirming the benefit of a higher field strength [37]. Liu et al. (2025) also demonstrated that increasing the applied voltage significantly enhanced erythrosine decolorization in a DBD system [38]. For dye pollutants, Huang et al. (2024) systematically evaluated voltage, frequency, and treatment time in a plasma bubbling array for methyl violet, finding that removal efficiency increased with voltage, and that voltage–frequency interactions strongly affected performance (RSM analysis) [39]. Consistent with these observations, Belkessa et al. (2023) showed that increasing voltage promoted methyl orange degradation and decolorization in a DBD–persulfate system, further linking stronger electric fields to accelerated plasma chemistry [40]. Finally, the effects of solution volume and liquid flow rate were also investigated, and the corresponding kinetic trends (Figure S4a,b) closely matched research data, confirming the reliability of the adopted experimental configuration.

Overall, both the experimental results and the literature evidence highlight the need for a balanced voltage selection: the applied voltage must be sufficiently high to generate abundant reactive species and ensure rapid decolorization, yet not so high as to induce filamentary discharges, localized overheating, or excessive energy consumption. In this study, an input voltage of 12 kV provided this optimal balance, delivering strong dye removal efficiency while maintaining stable plasma operation. Accordingly, 12 kV was selected as the operating voltage for all subsequent experiments.

#### 3.2.3. Effect of Gas Flowrate

**Figure 9:**
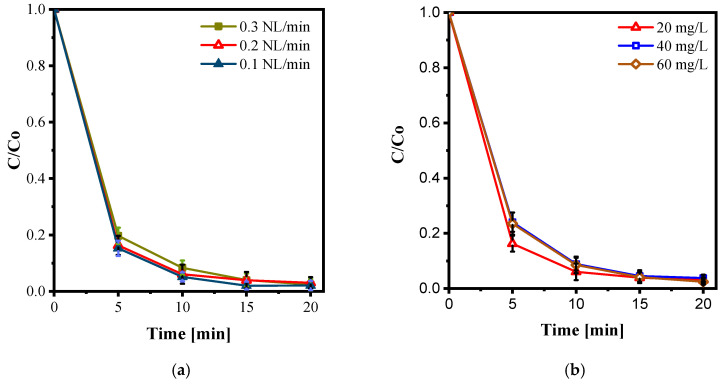
Effect of oxygen gas flow rate (**a**) and effect of Acid Yellow initial concentration (**b**), at 20 kHz, Gas: O_2_, LF: 84 mL m^−1^, Input voltage: 12 kV, Vol: 100 mL.

The gas flow rate determines the amount of oxygen introduced into the discharge and the residence time of gas molecules within the plasma region, thereby directly affecting the generation of reactive species (O, •OH, O_3_, H_2_O_2_) and their effective interaction with the liquid film. In this study, oxygen flow rates of 0.1, 0.2, and 0.3 NL·min^−1^ were investigated. As shown in Figure 9a, the 0.2 NL·min^−1^ condition yielded the best performance, achieving approximately 84% decolorization after 5 min and 96% after 10 min. In comparison, the 0.1 NL·min^−1^ and 0.3 NL·min^−1^ conditions resulted in slightly lower removals—around 82% and 95%, and 80% and 93% at 5 and 10 min, respectively.

These results indicate the existence of an optimal flow rate. At low flow (0.1 NL·min^−1^), oxygen availability limits radical formation, while at excessive flow (0.3 NL·min^−1^), the gas passes through the discharge region too rapidly, decreasing residence time, electron–molecule collisions, and consequently reactive oxygen species generation. Therefore, 0.2 NL·min^−1^ was selected as the optimal operating flow rate for the remainder of this study.

This optimization is fully consistent with the recent literature. Kim et al. (2024) compared two DBD modules for MB decolorization and explicitly varied the air flow rate, showing that changes in gas flow significantly affect ozone and ROS generation, as well as decolorization kinetics. Each reactor exhibited an optimal flow range (30 vs. 80 L·min^−1^), confirming that higher flow does not necessarily yield better performance [41]. Similarly, Nawaz et al. (2019) investigated O_2_ flow rate in a DBD water reactor for nitrobenzene decolorization and observed the same trend: an intermediate flow provided the highest removal efficiency, whereas both insufficient and excessive flows reduced performance [42]. Broader reviews of non-thermal plasma for water treatment consistently emphasize that gas type and flow rate are dominant operating variables, underscoring the importance of optimizing rather than maximizing the flow [43]. Reactor-scale studies further clarify this mechanism: in gas–liquid DDBD configurations, variations in flow rate alter gas–liquid contact patterns and discharge stability, leading to a practical optimum for pollutant removal [44]. Similarly, investigations of DBD systems for groundwater Fe/Mn removal demonstrated that excessive gas flow can shorten ionization time and lower energy utilization efficiency—consistent with the present observation that overly high flow reduces effective plasma chemistry despite increased gas throughput [45]. Finally, recent combined experimental and simulation studies on plasma dye decolorization confirmed that the effect of gas flow is multifaceted: while higher flow increases species supply, it simultaneously reduces residence time and reactive collisions, reinforcing the existence of an optimal flow range [38].

#### 3.2.4. Effect of AY36 Initial Concentration

The initial dye concentration determines the pollutant load relative to the flux of plasma-generated reactive species and therefore has a strong influence on decolorization kinetics. Figure 9b shows AY36 decolorization at three initial concentrations: 20, 40, and 60 mg/L. At 20 mg/L, removal was rapid, reaching 85% after 5 min and approximately 97% after 10 min. The decolorization rate decreased as the initial concentration increased, with slightly slower decay observed at 40 and 60 mg/L. Although nearly complete decolorization was achieved within 20 min for all concentrations, the apparent reaction rate was clearly highest at 20 mg/L. This behavior can be attributed to the competition among dye molecules for a fixed supply of reactive oxygen and nitrogen species generated by the plasma. As the initial concentration increases, more pollutant molecules share the same amount of radicals, leading to slower apparent kinetics, particularly in the early stages of the process. This trend is widely reported in the literature. Wu et al. (2019) explicitly varied the initial MB concentration in a DBD reactor and observed a decrease in decolorization rate with increasing C_0_, attributing the effect to a constant radical generation rate at the gas–liquid interface [36]. Similarly, Yao et al. (2023) investigated a gas–liquid hybrid DDBD system and found that increasing C_0_ reduced the apparent decolorization rate, in line with changes in air flow, pH, and applied voltage [44]. Consistent results were also reported by Kumar et al. (2022) for Acid Blue 25 decolorization using a cold plasma jet, where removal efficiency was higher at 25 mg·L^−1^ than at 50 mg·L^−1^ [46].

Based on these findings, an initial dye concentration of 20 mg/L was selected as the optimal condition for subsequent experiments. This concentration ensures fast decolorization kinetics (approaching complete decolorization within approximately 10 min) while remaining representative of real wastewater, which typically contains dye levels in the range of several tens of mg·L^−1^ (reported values around 65 mg·L^−1^ in raw effluents) [2].

### 3.3. Plasma and CeO_2_ Synergistic Effect Results

#### 3.3.1. Results of Experiments with Acid Yellow 36 Solution

**Figure 10:**
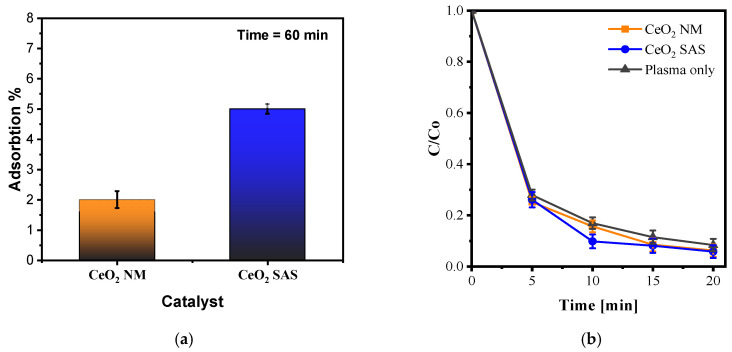
Plasma-off adsorption tests (**a**) and Effect of Ceria on AY36 decolorization (**b**) at optimized plasma parameters at 20 kHz, LF: 84 mL m^−1^, Q_O2_: 0.2 NL m^−1^; Input Voltage: 12 kV, Vol: 100 mL, CeO_2_: 0.05 g/L, AY36 initial concentration: 20 mg/L.

The first experiments with CeO_2_ catalysts (NM and SAS) were performed to evaluate the adsorption of AY36 on the catalyst surface prior to plasma exposure. As shown in Figure 10a, the dye adsorption was lower than 6% under the investigated conditions for both the catalytic materials. These results confirm that the rapid decolorization observed during plasma operation cannot be ascribed to adsorption alone, consistent with research recommendations to perform dark-control tests in plasma-catalysis studies to decouple physical uptake from plasma-induced reactions [47]. The negligible adsorption of AY36 on both CeO_2_ catalysts can be explained by their surface charge characteristics. CeO_2_ typically exhibits an isoelectric point around pH 6–7; below this range, the surface is positively charged, favoring adsorption of anionic dyes such as AY36, whereas above it, the surface becomes negatively charged, promoting interaction with cationic dyes such as CV [48]. Under the spontaneous pH conditions of our experiments (pH ≈ 6.4), the CeO_2_ surface carried a negative charge, leading to electrostatic repulsion with the anionic AY36 molecules. This charge mismatch explains the absence of significant dye adsorption and the nearly constant C/C_0_ values observed in dark conditions, in full agreement with the ζ-potential results discussed in Section 3.1.1.

Figure 10b compares three cases under the optimized plasma conditions: plasma only, plasma + CeO_2_ NM, and plasma + CeO_2_ SAS. The decolorization curves are nearly overlapping. After 5 min of treatment, the decolorization reached approximately 72% (plasma only), 73% (plasma + CeO_2_ NM), and 75% (plasma + CeO_2_ SAS); after 10 min, about 87%, 84%, and 83%, respectively. By 20 min, all cases converged around 93%. A pseudo–first-order fit in the 0–10 min range gave apparent rate constants of ≈0.20 min^−1^ (plasma), 0.18 min^−1^ (CeO_2_ NM), and 0.23 min^−1^ (CeO_2_ SAS). Hence, adding CeO_2_ did not produce a significant or consistent improvement for AY36 decolorization: the SAS catalyst showed only a minor acceleration at early times, which disappeared as the reaction progressed.

This outcome can be rationalized by electrostatic considerations at the liquid–solid interface. AY36 (Acid Yellow 36, also known as Metanil Yellow) is an anionic azo dye bearing sulfonate groups, with a visible absorption maximum near 435–436 nm, consistent with our UV–Vis monitoring [49]. Under the spontaneous pH of the reaction medium (pH ≈ 6.4), the CeO_2_ surface is slightly negatively charged, close to its isoelectric point. Recent studies report the IEP of CeO_2_ nanoparticles around pH 6.9–7.2, and comprehensive reviews of oxide surface charging confirm that ceria becomes neutral or weakly negative near neutral pH (Figure 4b) [50]. Consequently, the anionic AY36 molecules experience electrostatic repulsion from both CeO_2_ surfaces, which limits pre-adsorption and keeps most dye molecules in the bulk liquid. As established in the literature, dye adsorption and initial kinetic rates on oxide catalysts are strongly governed by surface charge relative to the IEP: when |ζ| is low near the IEP, electrostatic driving forces are weak and surface uptake is suppressed [51]. This interpretation is fully consistent with our tests in the absence of plasma exposure, confirming that the rapid decolorization observed during plasma exposure arises primarily from bulk plasma chemistry rather than catalyst-mediated interfacial processes.

In plasma–catalyst systems, significant synergy generally requires two simultaneous conditions: (i) efficient pollutant–catalyst contact through adsorption and (ii) the presence of catalytically active sites (e.g., Ce^3+^/Ce^4+^ redox pairs and oxygen vacancies) that can convert long-lived oxidants from the plasma (H_2_O_2_, O_3_) into highly reactive radicals (•OH, O). Reviews and case studies emphasize that when interfacial contact is limited, the synergy remains weak—precisely what is observed here for AY36 [47,52]. For CeO_2_ specifically, recent plasma-catalysis studies have shown that oxygen vacancies enhance MB and other dye decolorization by promoting H_2_O_2_ → •OH conversion; however, such benefits are most evident for cationic dyes that adsorb readily onto the negatively charged surface, not for anionic species that are repelled [53].

The DLS results (Figure 4a) help explain the slight early-time advantage of the SAS catalyst: CeO_2_ SAS displays a narrower sub-micron hydrodynamic size distribution, indicating better dispersion and a larger accessible surface area than the NM powder. Nevertheless, since AY36 is electrostatically repelled at pH ≈ 6.4, the increased area does not translate into stronger plasma–catalyst coupling. Under these near-neutral conditions, the charge mismatch (anionic AY36 vs. slightly negative CeO_2_) suppresses adsorption and thus limits synergistic effects. The literature suggests two possible strategies to enhance synergy with anionic dyes: operating below the IEP to render the CeO_2_ surface positively charged, or modifying the surface (e.g., with cationic coatings) to promote dye uptake—both approaches aiming to improve interfacial contact and activate CeO_2_ defect chemistry [51].

Since our goal here was to maintain the optimized neutral operating window, we proceeded instead to test cationic dyes (CV and MB), which are electrostatically attracted to the slightly negative CeO_2_ surface and are therefore more likely to exhibit clear plasma–catalyst synergy [54].

#### 3.3.2. Results of Experiments with Crystal Violet Solution

**Figure 11:**
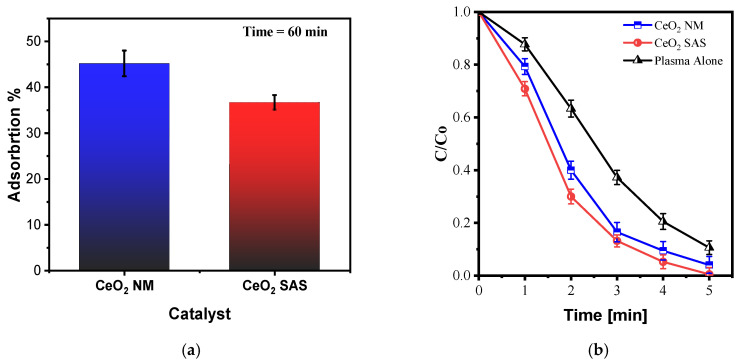
Plasma-off adsorption tests (**a**) and Effect of Ceria on CV decolorization (**b**) at optimized plasma parameters, 20 kHz, LF: 84 mL m^−1^, Q_O2_: 0.2 NL m^−1^, Input Voltage: 12 kV, Vol: 100 mL, CeO_2_: 0.05 g/L, CV initial concentration: 20 mg/L.

Figure 11a (plasma off) shows that CV exhibits strong adsorption on both CeO_2_ catalysts. After 60 min under dark conditions, the dye removal reached approximately 45% for CeO_2_ NM and 30% for CeO_2_ SAS, confirming a high affinity of the cationic dye for the negatively charged ceria surfaces.

These findings indicate a stronger interaction between CV and the catalyst surface. The ζ-potential profiles (Figure 4b) explain this behavior. At pH ≈ 6–7, both CeO_2_ samples are negatively charged (approximately −20 to −30 mV), with the commercial powder (CeO_2_ NM) exhibiting slightly more negative values than the SAS-derived sample at each pH point. Since CV is a cationic dye, a more negative surface enhances electrostatic attraction, which is consistent with the higher dark adsorption observed for the CeO_2_ NM catalyst.

This interpretation aligns with recent studies demonstrating that the adsorption of cationic dyes increases as the ζ-potential of the adsorbent becomes more negative. For instance, CV uptake has been shown to rise as surface charge shifts toward more negative values in batch adsorption systems [55], and selective capture of cationic dyes has been directly correlated with negative ζ-potentials in framework and oxide adsorbents [56,57].

Figure 11b shows the decolorization kinetics of CV under the optimized plasma conditions for three cases: plasma only, plasma + CeO_2_ NM, and plasma + CeO_2_ SAS. The addition of ceria significantly enhanced the decolorization rate compared to plasma alone, with CeO_2_ SAS slightly outperforming the non-micronized catalyst. Complete decolorization was achieved within approximately 5 min with CeO_2_ SAS, whereas the plasma-only system required a longer time to reach the same level.

The superior performance of the SAS-processed catalyst can be attributed to both its morphological and electronic properties. The DLS results (Figure 4a) revealed that SAS-CeO_2_ forms a narrow sub-micron particle population with improved dispersion and larger accessible surface area in aqueous suspension. Moreover, SAS processing typically introduces a higher density of structural defects and Ce^3+^/Vo sites (Figure 6), which are known to act as active centers for the activation of long-lived plasma oxidants such as O_3_ and H_2_O_2_, generating short-lived radicals (•OH and O) that accelerate dye decolorization.

This interpretation agrees with recent studies. Quezada-Urbina et al. (2024) reported that plasma-modified CeO_2_ with increased oxygen-vacancy density achieved faster MB decolorization (≈90% in 30 min) compared with untreated ceria [53]. Likewise, Aggelopoulos et al. (2024) emphasized in their review that vacancy-rich CeO_2_ is among the most effective plasma catalysts when adequate pollutant–surface contact is ensured [58]. In line with these findings, Vaiano et al. (2022) demonstrated that coupling non-thermal plasma with ceria-based catalysts markedly improves dye removal compared to plasma alone, confirming the synergistic behavior observed here for cationic dyes under O_2_-fed DBD operation [13].

Given the slightly higher activity and better dispersion of the SAS-micronized catalyst, CeO_2_ SAS was selected for the subsequent experimental investigations.

#### 3.3.3. Scavenger Test

**Figure 12:**
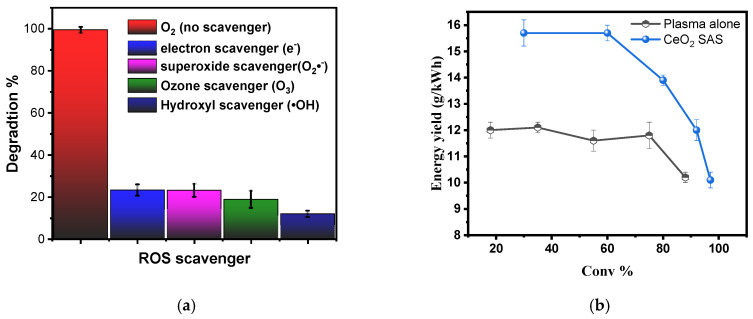
Effect of radical scavenger on CV decolorization (**a**) and energy yield (**b**) for different concentrations, at optimized plasma parameters, 20 kHz, LF: 84 mL m^−1^, Input voltage: 12 kV, Vol: 100 mL, CeO_2_ SAS: 0.05 g/L. Qgas: 0.2 NL m^−1^, CV initial concentration: 20 mg/L.

Figure 12a shows the effect of selective scavengers on CV decolorization under the optimized plasma conditions. In the absence of scavengers, the dye underwent rapid decolorization. The addition of isopropyl alcohol (IPA), a well-known •OH radical scavenger, almost completely suppressed the decolorization process, indicating that hydroxyl radicals play a dominant role. The introduction of p-benzoquinone (BQ, superoxide scavenger) and K_2_Cr_2_O_7_ (hydrated-electron scavenger) also led to a marked reduction in decolorization rate, while replacing oxygen with helium—thereby minimizing ozone formation—resulted in a much slower decay. Collectively, these results establish the following reactivity hierarchy: •OH ≫ O_3_/O_2_•^−^/e^−^, demonstrating that hydroxyl radicals are the primary oxidants responsible for early-stage CV decolorization, whereas ozone, superoxide, and solvated electrons provide secondary but significant contributions.

This interpretation aligns well with the plasma–water chemistry literature. Quantitative spin-trapping and dosimetry studies consistently show •OH as the most reactive and abundant oxidant in non-thermal plasma water treatment, consistent with the strong inhibition by IPA observed here [59]. The use of p-benzoquinone to probe O_2_•^−^ is standard practice; kinetic compilations report diffusion-controlled rate constants for BQ with O_2_•^−^ and note that BQ can also intercept •OH and electrons, explaining the substantial but not total inhibition we observed [60]. Potassium dichromate acts as a classical scavenger of hydrated electrons, and pulse-radiolysis studies confirm its fast electron-capture kinetics, in agreement with the slower decay observed when K_2_Cr_2_O_7_ was added [61].

Ozone formation in the discharge follows the three-body recombination pathway:

O + O_2_ + M → O_3_ + M

where atomic oxygen (O) originates from electron-impact dissociation of O_2_. When oxygen is replaced with helium, O_3_ generation collapses, since the recombination route is suppressed. The much slower dye decolorization observed under helium feeding therefore confirms that ozone—either as a direct oxidant or through its well-known aqueous ozonation chemistry leading to •OH formation—plays an important supporting role [62].

Under oxygen-fed DBD conditions, energetic electrons and excited oxygen species drive a network of plasma–liquid reactions that produce the reactive species probed in the scavenger tests:

(i) Electron-impact on water:

$$e^{-}+H_{2}O\to ∙OH+H∙+e^{-}$$

and related dissociative excitation channels are well characterized for e^−^–H_2_O collisions [63,64]. This reaction constitutes a primary source of hydroxyl radicals directly in the plasma–liquid interface.

(ii) Excited oxygen reacting with water:

O(D1)+H2O→2∙OH

The metastable O(^1^D) species arises from e^−^–O_2_ interactions or from O_3_ photolysis and afterglow reactions, and reacts rapidly with water to yield •OH radicals [65].

(iii) Ozone-initiated OH in water (Weiss mechanism):

Dissolved O_3_ triggers chain reactions involving the HO_2_^−^/O_2_•^−^ equilibrium:

$$O_{3}+HO_{2}^{-}\to ∙OH+O_{2}^{∙-}+O_{2}$$

Pulse-radiolysis and ozonation studies have documented this pathway as a key contributor to aqueous •OH production [66].

(iv) Superoxide formation and equilibria:

$$O_{2}+e_{aq}^{-}\to O_{2}^{∙-}$$

$$O_{2}^{∙-}+H^{+}⇌HO_{2}^{∙}(pK_{a}\approx 4.8)$$

Superoxide and its protonated form participate in the ozonation chain, disproportionate to H_2_O_2_, and act as precursors for further •OH generation. Scavenging O_2_•^−^ with p-benzoquinone (BQ) markedly slows CV decolorization, consistent with its role as a chain carrier.

(v) Hydrated electrons $e_{aq}^{-}$:

Interfacial solvation of electrons is a hallmark of plasma–liquid systems [67]. These electrons are rapidly captured by electron acceptors such as Cr(VI); their removal by K_2_Cr_2_O_7_ suppresses the formation of O_2_•^−^ and downstream oxidants (H_2_O_2_, •OH), in full agreement with the observed inhibition in Figure 12a.

Collectively, these pathways explain the scavenger effects observed experimentally:

IPA quenches •OH, almost completely halting decolorization, confirming that hydroxyl radicals are the dominant oxidants.

BQ scavenges O_2_•^−^ and, to a lesser extent, •OH and e^−^, leading to partial inhibition.

K_2_Cr_2_O_7_ captures hydrated electrons, reducing radical chain propagation and slowing the process.

Helium feeding suppresses O_3_ formation, confirming that ozone contributes—directly or indirectly via •OH generation—to the overall reactivity [68].

Overall, the experimental scavenger ranking (•OH ≫ O_3_/O_2_•^−^/e^−^) and the mechanistic framework are fully consistent: hydroxyl radicals are the principal oxidants driving fast CV decolorization, while ozone (via direct oxidation and •OH production), superoxide (as a chain carrier and H_2_O_2_ precursor), and hydrated electrons (as reductive initiators of O_2_•^−^ chemistry) play important secondary roles under oxygen-fed DBD operation. This hierarchy aligns with detailed plasma–liquid reviews and quantitative OH-dosimetry studies [59,69].

#### 3.3.4. Energy Efficiency

Energy efficiency is a key performance metric for plasma-based processes, as it quantifies the amount of pollutant degraded per unit of electrical energy consumed. In plasma-assisted oxidation systems, this parameter enables a direct comparison between catalytic and non-catalytic configurations, revealing how catalyst design and operating conditions affect the overall energy demand of the process.

As shown in Figure 12b, at a constant power input of 23 W, the SAS–CeO_2_ catalyst consistently achieved higher energy efficiency than both plasma alone and the non-micronized CeO_2_ (Figure S6b). Within the 30–60% conversion range, the energy yield (Y) remained around 15–16 g·kWh^−1^ for SAS–CeO_2_, compared with approximately 11–12 g·kWh^−1^ for plasma alone—representing a 40–50% improvement. At higher conversion levels, Y decreased for all cases, as expected from radical depletion and mass-transfer limitations; however, SAS–CeO_2_ still reached ≈10 g·kWh^−1^, maintaining a clear advantage. This superior energy performance is consistent with the combined effects of dye pre-adsorption and vacancy-assisted •OH generation at the ceria–solution interface.

These trends are in line with previous reports on DBD-based dye abatement, where the energy yield is widely employed as a benchmark. In a recent study, it was reported that the treatment process operated at a power of 5.54 W and achieved an energy yield of 28.51 g kW^−1^ h^−1^ at 50% decolorization, with an electrical energy per order of 2.91 kWh m^−3^ for Amx removal [70]. By definition, Y represents the mass of pollutant degraded per unit of energy input (g·kWh^−1^), and typically increases with initial concentration while decreasing at prolonged treatment times due to kinetic and scavenging limitations. For example, Wu et al. (2019) reported MB energy yields up to ~9.3 g·kWh^−1^ in a diffuser/packed-bed DBD reactor [71], whereas Zaffar et al. (2025) compared two atmospheric plasma jet designs and found Y values between 2.1 and 2.9 g·kWh^−1^ depending on reactor configuration [72]. The present results therefore position SAS–CeO_2_ among the most energy-efficient plasma-assisted systems reported for dye decolorization.

#### 3.3.5. Degradation Efficiency in the Mixed Dye Solution (CV and MB)

**Figure 13:**
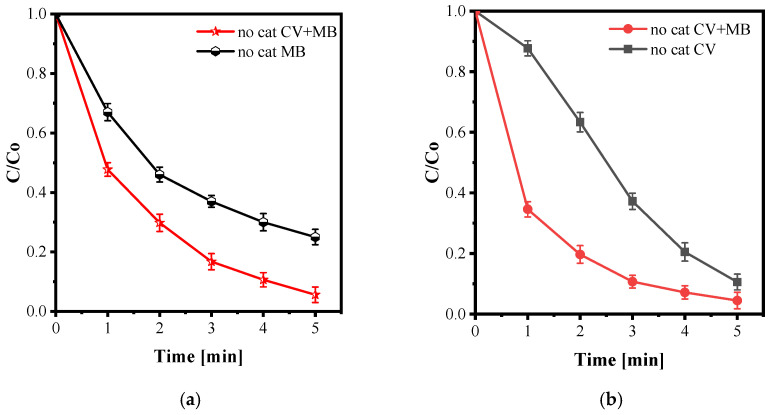
(**a**) decolorization of MB and (**b**) decolorization of CV in the mixed-dye solution under optimized plasma conditions (20 kHz, O_2_ flow rate: 0.2 NL·min^−1^, liquid flow: 84 mL min^−1^, input voltage: 12 kV, solution volume: 100 mL; initial concentrations: CV = 20 mg/L, MB = 20 mg/L).

Using the optimized plasma settings, the decolorization behaviour of a mixed solution containing CV and MB was investigated. Interestingly, the mixed-dye solution degraded more rapidly than either dye treated individually. As shown in Figure 13a,b, a clear synergistic effect emerges in the mixed system when plasma is applied without any catalyst.

A similar phenomenon has been reported in the literature for mixtures of organic pollutants exposed to non-thermal plasma. Liu et al. (2024) observed that the coexistence of multiple compounds can lower the overall redox barriers and facilitate electron-transfer pathways, thereby accelerating decolorization compared to the isolated treatment of each pollutant [73].

Reviews on cold plasma for water treatment have emphasized that the composition of the reaction matrix can significantly alter the ROS network and the associated reaction pathways. In some cases, the coexistence of multiple pollutants enhances overall decolorization efficiency by promoting synergistic radical reactions—consistent with our observations here [74,75].

**Figure 14:**
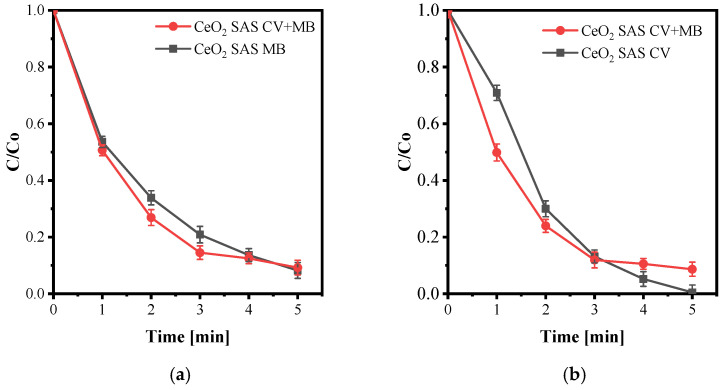
MB degradation and decolorization with mixed dye in the presence of catalyst (**a**), CV decolorization with mixed dye in the presence of catalyst (**b**), at optimized plasma parameters, 20 kHz, Q_O2_: 0.2 NL·min^−1^, MB initial concentration: 20 mg/L, CV initial concentration: 20 mg/L, LF: 84 mL m^−1^, Input: 12 kV, Vol: 100 mL, CeO_2_: 0.05 g/L.

In the presence of the CeO_2_ SAS catalyst, both dyes (CV and MB) degraded more rapidly than with plasma alone (Figure 14). For MB, the decolorization profiles (Figure 14a) were nearly identical for the single- and mixed-dye solutions when the catalyst was present, indicating that the presence of CV did not significantly influence MB removal. In contrast, for CV (Figure 14b), the mixed-dye solution exhibited slightly faster decolorization during the initial stage, but did not achieve complete decolorization within 5 min, unlike the single-dye system, which reached nearly 100% removal.

This behavior can be explained by competitive adsorption between the two cationic dyes on the negatively charged CeO_2_ surface at pH ≈ 6.4. Both CV and MB molecules accumulate at the catalyst–liquid interface, where they compete for active sites and reactive interfacial species. Li et al. systematically investigated binary MB–CV systems and demonstrated that such competition alters both adsorption capacity and apparent kinetics compared with single-dye experiments—precisely the trend observed here for CeO_2_ SAS [76]. Similar effects have been reported for MB and CV in other binary and ternary systems, where coexisting dyes reduce adsorption capacity and slow decolorization relative to individual treatments [77].

In photocatalytic co- decolorization studies, Foroutan et al. likewise observed that simultaneous treatment of CV and MB modifies the reaction kinetics and optimal operating conditions compared with single-dye runs, due to competition for adsorption sites and shared radical flux [78].

## 4. Conclusions

This work demonstrated the optimization of a coaxial falling-film DBD reactor and its coupling with ceria catalysts for the decolorization of dyes. The systematic investigation of plasma parameters (feeding gas, voltage, flow rate, and initial dye concentration) identified oxygen feeding, 12 kV input voltage, 0.2 NL·min^−1^ gas flow, and 20 mg·L^−1^ initial concentration as the optimal operating window for efficient plasma oxidation.

Comparative tests between non-micronized CeO_2_ and CeO_2_ synthesized via supercritical antisolvent (SAS) precipitation revealed that SAS-derived catalysts consistently exhibited superior performance, particularly for cationic dyes such as Crystal Violet (CV) and Methylene Blue (MB). The SAS process produced ceria with narrow sub-micron particle distribution (DLS) and uniform spherical morphology (SEM), improving aqueous dispersibility and plasma–catalyst contact. Raman analysis further confirmed a slightly higher oxygen-vacancy concentration in SAS–CeO_2_, supporting its enhanced redox activity.

The decolorization trends demonstrated that cationic dyes benefited from strong plasma–catalyst synergy, whereas the anionic dye Acid Yellow 36 (AY36) showed negligible improvement due to electrostatic repulsion at the natural pH (~6.4). ζ-potential analysis clarified this surface-charge dependence, establishing a mechanistic guideline: matching catalyst surface charge with dye charge is essential to unlock plasma–ceria synergy.

Scavenger experiments confirmed that hydroxyl radicals (•OH) are the dominant oxidizing species driving dye decolorization, with ozone, superoxide, and hydrated electrons acting as secondary contributors.

Beyond kinetics, coupling the reactor with SAS–CeO_2_ significantly improved energy efficiency, achieving 40–50% higher energy yield (≈15–16 g·kWh^−1^) compared to plasma alone under equivalent power input. Finally, tests on mixed-dye solutions (CV + MB) revealed plasma-induced synergy even without the catalyst, while in the presence of CeO_2_ SAS, both dyes degraded rapidly but exhibited mild competitive adsorption—consistent with binary dye interaction mechanisms reported in the literature.

Overall, this study confirms that micronization of CeO_2_ via SAS is an effective materials-engineering strategy to enhance plasma–catalyst synergy. The resulting system achieves faster decolorization, improved energy efficiency, and a tuneable, charge-dependent catalytic response. These findings provide a practical framework for designing plasma-assisted advanced oxidation processes tailored for real wastewater treatment.

The DBD reactor is modular and in principle scalable, but increasing throughput poses challenges in maintaining discharge stability and efficient gas–liquid contact. These limitations will need to be addressed through targeted reactor design and dedicated plasma diagnostics in future work.

## Figures and Tables

**Table 1:** Physical characteristics of CeO_2_ NM and CeO_2_ SAS.

Photocatalyst	Specific Surface Area (m^2^/g)	Hydrodynamic Diameter (nm)	Polydisperse Index (-)
CeO_2_ NM	46	618	0.6620
CeO_2_ SAS	47	387	0.6470

**Table 2:** O_v_/F_2g_ intensity ratio and Band Gap of tested photocatalyst.

Photocatalyst	I_OH_/I_Ce-O_ (-)	O_v_/F_2g_ Intensity Ratio (-)	Band Gap (eV)
CeO_2_ NM	1.77	0.0260	2.60
CeO_2_ SAS	2.62	0.0280	2.68

**Table 3:** First order kinetic constant and half-life time for decolorization of AY36 on different gas type.

Plasma Type	k_d_ (min^−1^)	t12 (min)
Air	0.067	10.3
Oxygen	0.135	5.1

**Table 4:** First order kinetic constant and half-life time for decolorization of AY36 on different input voltage.

Input Voltage	k_d_ (min^−1^)	t12 (min)
10 kV	0.110	6.32
12 kV	0.094	7.36
16 kV	0.128	5.43

## Data Availability

The original contributions presented in this study are included in this article/Supplementary Materials. Further inquiries can be directed to the corresponding authors.

## Supplementary Materials

The following supporting information can be downloaded at: https://www.mdpi.com/article/10.3390/nano15231831/s1, Figure S1: Real-time picture of DBD reactor in working condition; Figure S2. (a) voltage and current wave form (b) Lissajous figure of DBD reactor; Figure S3. (a) UV-Vis spectrum of MB and AY 36; Figure S4. Effect of (a) volume and (b) liquid flow rate on the decolorization; Figure S5. (a) Dark adsorption; (b) Plasma-ceria synergy; Figure S6. (a) Energy yield on different concentration; (b) energy yield with and without Catalyst. Figure S7. Emission spectrum of oxygen plasma only water and water with oxygen. Figure S8. XRD spectra for CeO_2_ NM and CeO_2_ SAS samples.
