# Supplementary material for: Non-Thermal Plasma-Driven Degradation of Organic Dyes Using CeO2 Prepared by Supercritical Antisolvent Precipitation
Source: Nanomaterials (Basel). 2025 Dec 4;15(23):1831. doi: 10.3390/nano15231831 (PMC12693223; doi:10.3390/nano15231831)
Supplement: Supplementary file 1 [file nanomaterials-15-01831-s001.zip › nanomaterials-4012710-supplementary.pdf]

Supplementary Information for

Non-Thermal Plasma-Driven Degradation of Organic Dyes Using CeO_2 Prepared by Supercritical Antisolvent Precipitation

Qayam Ud Din, Maria Chiara Iannaco *, Iolanda De Marco, Vincenzo Vaiano and Giuseppina Iervolino *

1. DBD reactor in working condition

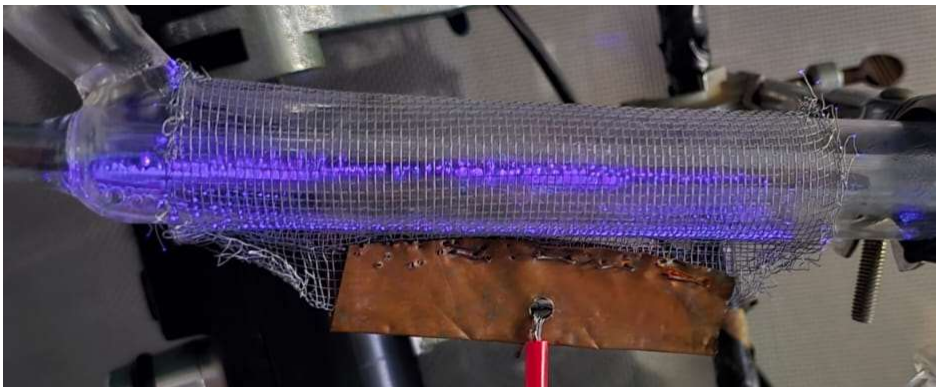

Figure S1. Real time picture of DBD reactor in working condition

2. Electrical properties of DBD reactor:

Figure S2a. Voltage–current waveform of the coaxial falling-film DBD reactor at 12 kV and 20 kHz. The sinusoidal voltage profile (black) corresponds with the discharge current (red), where the sharp current spikes represent the formation of micro-discharges typical of dielectric barrier discharge plasma.

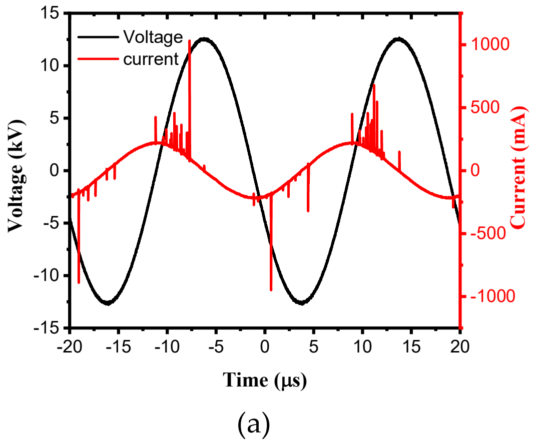

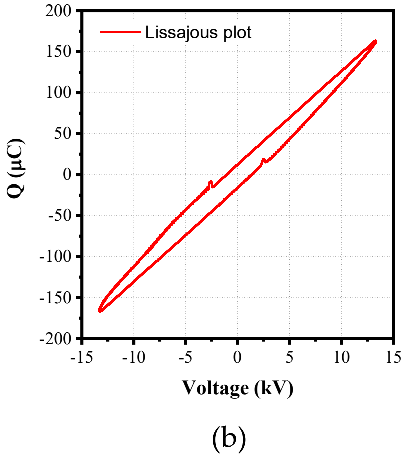

Figure S2. (a) voltage and current wave form (b) Lissajous figure of DBD reactor

These transient pulses are responsible for generating highly reactive species such as •OH, O, and O_3, which drive the degradation of organic pollutants. Similar current–voltage behaviours have been reported for DBD systems applied in wastewater treatment, and **Figure S2b.** Lissajous figure of the DBD reactor showing the Q–V relationship. The enclosed area corresponds to the energy dissipated per cycle, from which the average plasma power was calculated as ~23 W.

3. Absorbance spectrum of methylene blue and acid yellow 36

Figure S3 represent UV–Vis absorbance spectra of (a) methylene blue and (b) acid yellow 36 during plasma treatment at different time intervals. The progressive decrease in the main absorption peaks (~664 nm for methylene blue and ~430 nm for acid yellow 36) confirms the effective breakdown of the dye chromophores by reactive plasma species.

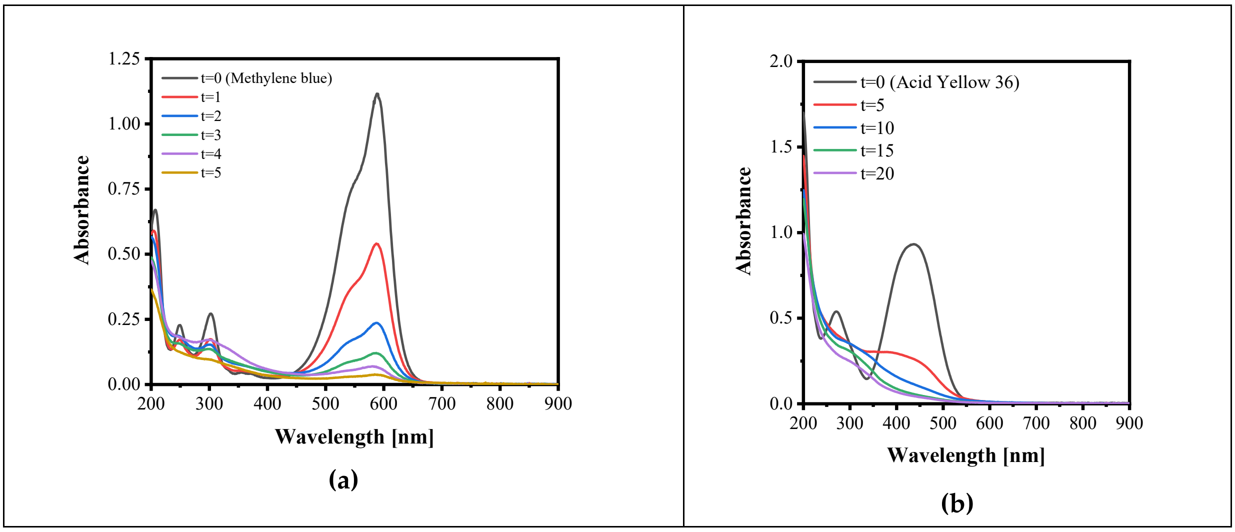

Figure S3. (a) UV-Vis spectrum of MB and AY 36

4. Effect of sample volume and liquid flow rate on the degradation of AY36

Figure S4. Effect of (a) solution volume and (b) liquid flow rate on the degradation efficiency of dyes in the coaxial falling-film DBD reactor. Smaller solution volumes (50–100 mL) exhibited faster degradation compared to larger volumes (150 mL), due to higher energy density per unit volume and enhanced plasma–liquid interaction. Similarly, higher flow rates (84 mL/min, 200 rpm) improved dye removal compared to lower flow rates (42 mL/min, 100

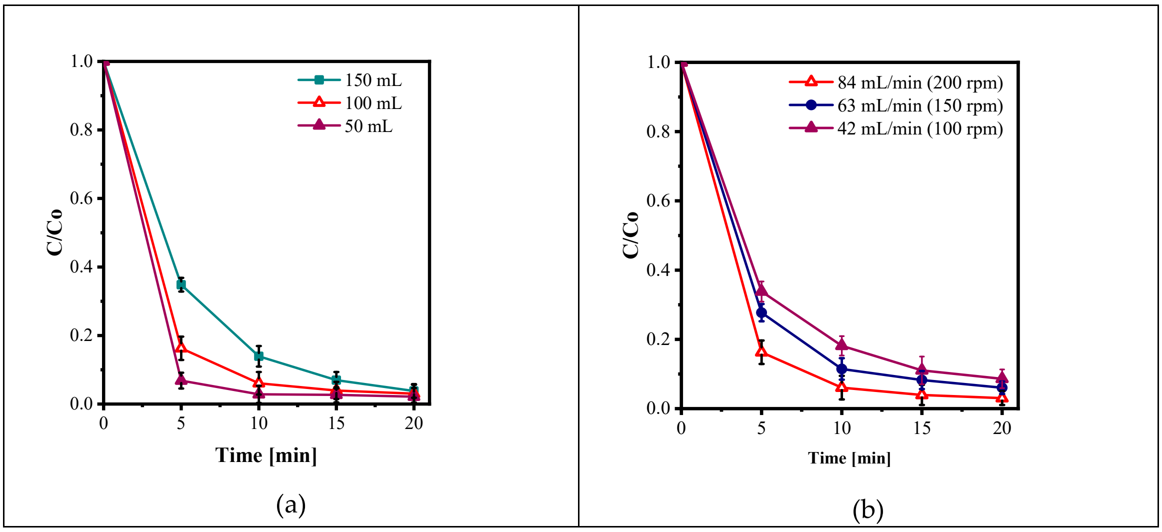

Figure S4. Effect of (a) volume and (b) liquid flow rate on the degradation

rpm), as increased liquid renewal at the plasma interface enhances mass transfer and contact with reactive plasma species.

5. Methylene Blue (MB): dark adsorption and plasma–ceria synergy at optimized conditions

Dartk adsorption:

Figure S5a shows that Methylene Blue (MB) adsorbs modestly on both ceria powders in the dark. Within 10 min, the concentration ratio decreases to about $C/C_0 \approx 0.86$ for the CeO_2 NM and ≈0.90 for the CeO_2 SAS, and then remains nearly constant over 60 min. This behaviour is consistent with electrostatics: at our working pH (~6.4), ceria surfaces are already slightly negative (near their isoelectric/point-of-zero-charge window), so the cationic MB molecule experiences net attraction and can pre-adsorb, albeit not as strongly as Crystal Violet.

Plasma-ceria synergy:

Figure S5b compares plasma only with plasma + CeO_2 at the optimized parameters for MB. The catalysts clearly accelerate decolorization relative to plasma alone. A pseudo–first-order fit over 0–3 min gives apparent rate constants of $k_{plasma} \approx 0.37\ min^{-}$, $k_{NO\text{-}SAS} \approx 0.49\ min^{-}$, and $k_{SAS} \approx 0.62\ min^{-1}$. Thus, the enhancement ratios $k_{cat+plasma}/k_{plasma}$ are about 1.3× (CeO_2 NM) and 1.7× (CeO_2 SAS).

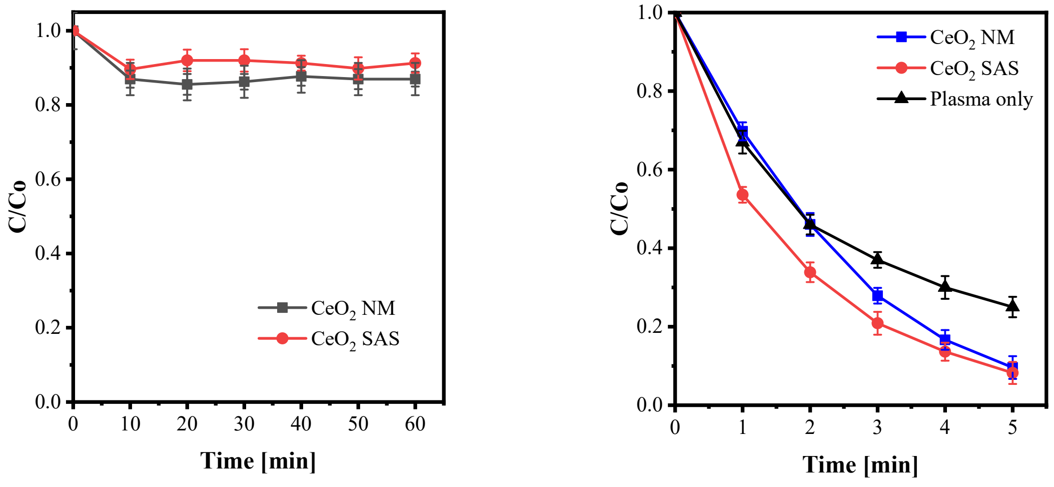

(a) (b)

Figure S5. (a) Dartk adsorption (b) Plasma-ceria synergy for MB

6. Methylene Energy yield for different concentrations:

Figure S6 shows that energy yield is higher at higher initial dye concentration: the 60 mg/L curve lies above 40 mg/L, which lies above 20 mg/L at the same conversion. This is expected because, for the same input power and time, more mass is removed when the starting

concentration is higher, so Y rises. Conversely, Y decreases as conversion approaches 100% for each series, reflecting slower late-stage kinetics (fewer reactant molecules and more by-products/oxygen-scavengers), so each additional percent of conversion costs more energy.

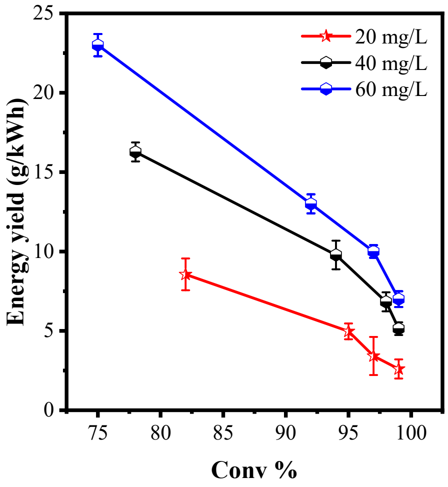

(a)

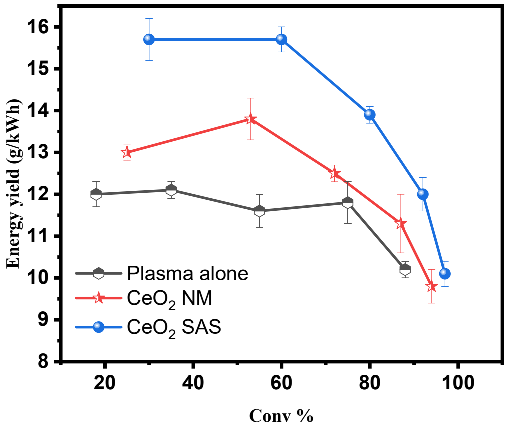

(b)

Figure S6. (a) energy yield on different concentration (b) energy yield with and without Catalyst

7. Optical emission spectroscopy (OES)

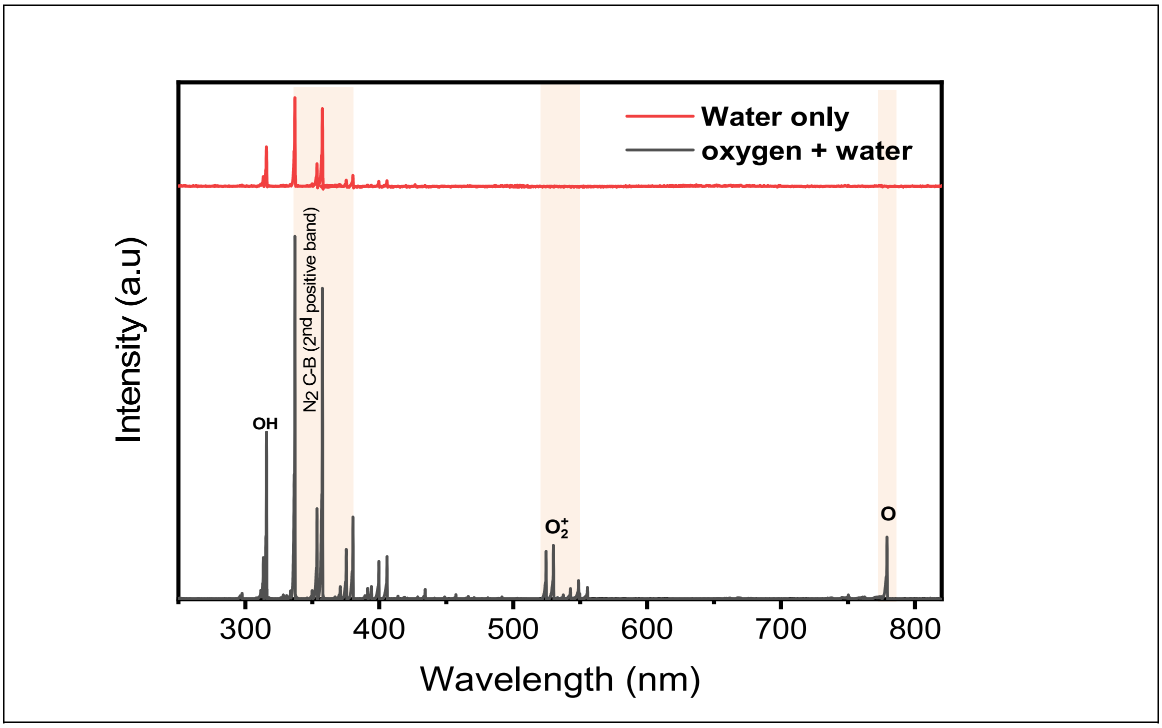

Figure S7. Emission spectrum of oxygen plasma only water and water with oxygen

XRD DIAGRAMM FOR THE TWO PREPARED CATAYSTS

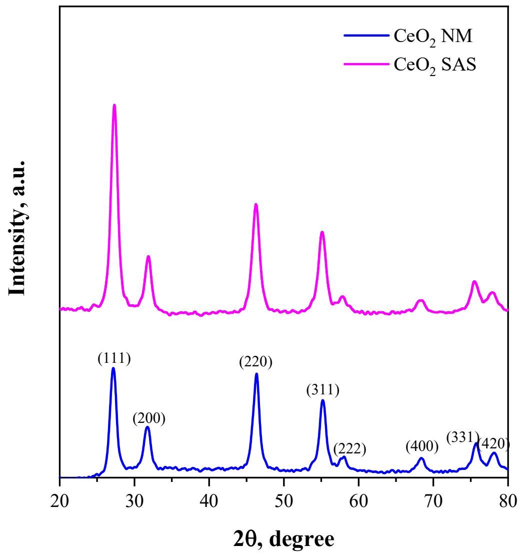

Figure S8. XRD spectra for CeO2 NM and CeO2 SAS samples

For both catalysts the crystallite size is about 9 nm.
